# Supplementary material for: Elucidating the active interaction mechanism of phytochemicals withanolide and withanoside derivatives with human serum albumin
Source: PLoS One. 2018 Nov 7;13(11):e0200053. doi: 10.1371/journal.pone.0200053 (PMC6221254; doi:10.1371/journal.pone.0200053)

**S1 Fig.** The profile of atomic fluctuations. Atomic fluctuations of unliganded HSA and HSA- Withanolide and Withanoside complexes to the active site amino acid residues present in the site I of HSA.


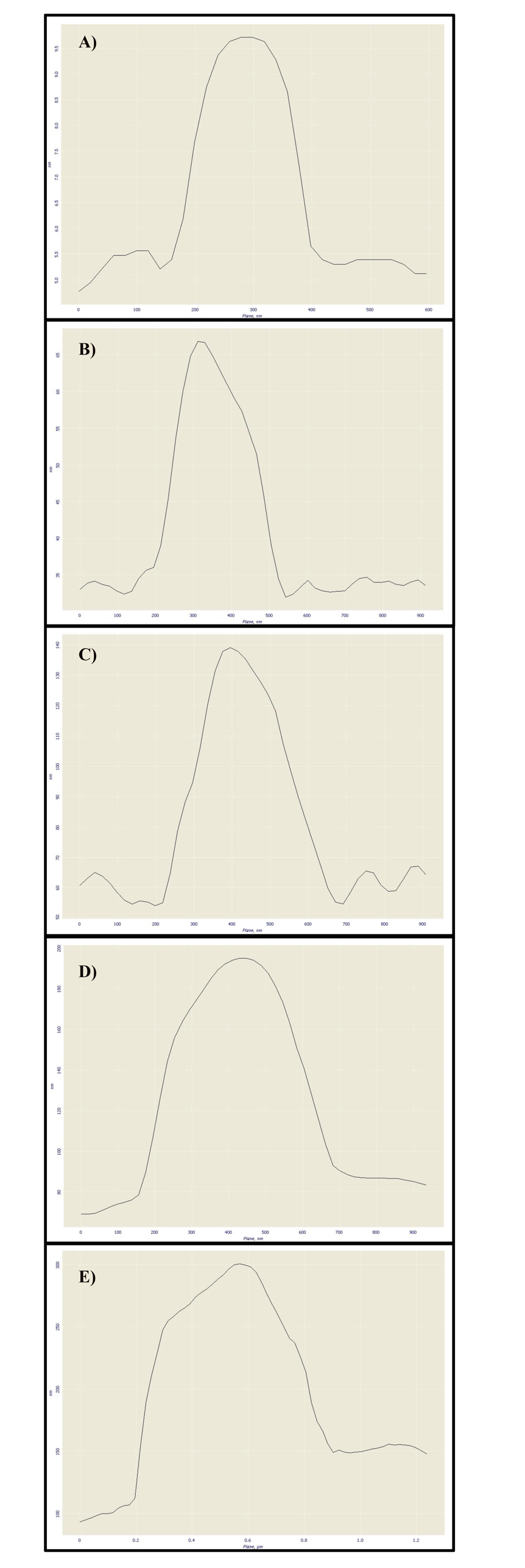

Supplement: S1 Fig — Atomic fluctuations of unliganded HSA and HSA- Withanolide and Withanoside complexes to the active site amino acid residues present in the site I of HSA. (DOCX) [file pone.0200053.s001.docx]
