# Supplementary material for: Elucidating the active interaction mechanism of phytochemicals withanolide and withanoside derivatives with human serum albumin
Source: PLoS One. 2018 Nov 7;13(11):e0200053. doi: 10.1371/journal.pone.0200053 (PMC6221254; doi:10.1371/journal.pone.0200053)

**S2 Fig**. The HSA-Withanoside derivative complexes at 10 ns simulations showed rigidity of residues in their respective domains


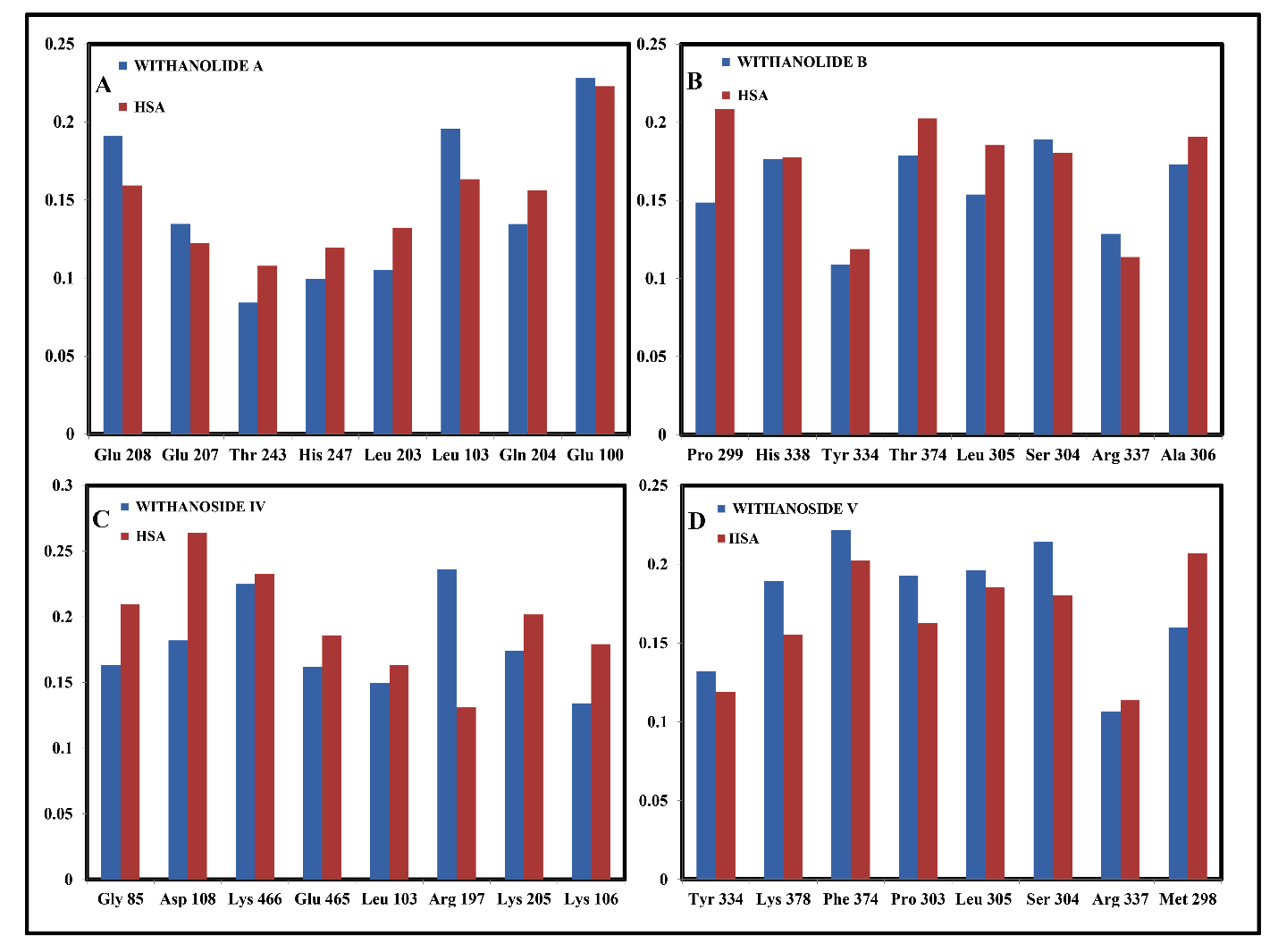

Supplement: S2 Fig — (DOCX) [file pone.0200053.s002.docx]
