# Supplementary material for: Elucidating the active interaction mechanism of phytochemicals withanolide and withanoside derivatives with human serum albumin
Source: PLoS One. 2018 Nov 7;13(11):e0200053. doi: 10.1371/journal.pone.0200053 (PMC6221254; doi:10.1371/journal.pone.0200053)
Supplement: S1 Table — (DOCX) [file pone.0200053.s003.docx]

**S1 Table**. This table shows percentage change in the secondary structure upon addition of different concentration of withanolide and withanoside drugs.

| **Secondary Structures (%)** | **α-Helix** | **β-Turn** | **Random Coil** |
| --- | --- | --- | --- |
| **HSA** | 58.40±2.5 | 13.1±0.62 | 16.30±0.82 |
| **HSA + withanolide A (2μM)** | 66.40±2.5 | 12.0±0.65 | 13.20±0.82 |
| **HSA + withanolide A (4μM)** | 67.50±2.43 | 11.8±0.62 | 12.30±0.90 |
| **HSA + withanolide A (6μM)** | 69.30±2.5 | 11.7±0.66 | 11.2±0.83 |
| **HSA + withanolide B (2μM)** | 66.91±2.63 | 12.0±0.62 | 13.2±0.82 |
| **HSA + withanolide B (4μM)** | 68.31±2.53 | 11.7±0.71 | 12.1±0.86 |
| **HSA + withanolide B (6μM)** | 70.54±2.5 | 10.3±0.62 | 11.8±0.80 |
| **HSA + withanoside IV(2μM)** | 57.8±2.47 | 13.3±0.64 | 16.3±0.90 |
| **HSA + withanoside IV(4μM)** | 64.9±2.56 | 13.1±0.63 | 16.7±0.87 |
| **HSA + withanoside IV(6μM)** | 66.8±2.5 | 12.8±0.62 | 17.8±0.83 |
| **HSA + withanoside V(2μM)** | 58.8±2.3 | 13±0.62 | 15.3±0.84 |
| **HSA + withanoside V(4μM)** | 60.7±2.4 | 12.8±0.71 | 16.7±0.85 |
| **HSA + withanoside V(6μM)** | 61.5±2.3 | 12.9±0.63 | 18.3±0.87 |
